# Gravity, Quantum Fields and Quantum Information: Problems with Classical Channel and Stochastic Theories

**DOI:** 10.3390/e24040490

**Published:** 2022-03-31

**Authors:** Charis Anastopoulos, Bei-Lok Hu

**Affiliations:** 1 Department of Physics, University of Patras, 26500 Patras, Greece; 2 Maryland Center for Fundamental Physics and Joint Quantum Institute, University of Maryland, College Park, MD 20742-4111, USA; blhu@umd.edu

**Keywords:** quantum information, quantum gravity, quantum fluctuations, alternative quantum theories, semi-classical theories

## Abstract

In recent years an increasing number of papers have attempted to mimic or supplant quantum field theory in discussions of issues related to gravity by the tools and through the perspective of quantum information theory, often in the context of alternative quantum theories. In this article, we point out three common problems in such treatments. First, we show that the notion of interactions mediated by an information channel is not, in general, equivalent to the treatment of interactions by quantum field theory. When used to describe gravity, this notion may lead to inconsistencies with general relativity. Second, we point out that in general one cannot replace a quantum field by a classical stochastic field, or mock up the effects of quantum fluctuations by that of classical stochastic sources (noises), because in so doing important quantum features such as coherence and entanglement will be left out. Third, we explain how under specific conditions semi-classical and stochastic theories indeed can be formulated from their quantum origins and play a role at certain regimes of interest.

## 1. Preamble

The rapid growth of quantum information science has led to the application of Quantum Information Theory (QIT) concepts to different branches of physics, from condensed matter to cosmology and quantum gravity. In particular, QIT concepts are used to formulate models for new physics, such as alternative quantum theories (AQT) or gravitational interactions. The key point we make in this paper is that these models must be structurally compared with our current best theories for the phenomena they purport to describe, namely, Quantum Field Theory (QFT) and General Relativity (GR). Even if these models have good motivations and their own logic, they must pass the scrutiny of these two well-tested theories or be embedded in a framework that relies on these proven theories. Failure to do so renders such models physically inconsistent and, hence, a priori implausible.

In this paper, we focus on three particular problems that commonly appear in such models. First, we show that the QIT notion of an information channel does not, in general, capture the idea of mediating fields in QFT. It is therefore misleading to use it as a basis for describing, for example, the gravitational interaction. Second, we argue that we cannot in general substitute quantum fluctuations in an unknown theory with classical stochastic noises. Third, we explain the proper way to derive the stochastic and semi-classical theories from their quantum origins.

## 2. Problem 1: Quantum Fields Misconstrued as Information Channels

### 2.1. Quantum Channels

A quantum channel is a map *C* that takes quantum states ${\hat{\rho }}_{e}$ on a Hilbert space $H_{A}$ (emitter) to quantum states $C[{\hat{\rho }}_{e}]$ on a Hilbert space $H_{B}$ (receiver). The map is completely positive and trace preserving.

Two types of quantum transmission are directly relevant: direct interaction channels and channels with a mediator.

- In a *direct interaction* channel, the emitter and receiver interact through a unitary map $\hat{U}$ on $H_{A}⊗H_{B}$. Then
  (1) $$\begin{array}{c} C\left[{\hat{\rho }}_{e}\right]=Tr_{H_{A}}\left(\hat{U}\left({\hat{\rho }}_{e}⊗{\hat{\rho }}_{0}\right){\hat{U}}^{†}\right), \end{array}$$
  where ${\hat{\rho }}_{0}$ is the initial state of the receiver. Usually $\hat{U}$ is generated by a Hamiltonian ${\hat{H}}_{A}+{\hat{H}}_{B}+{\hat{H}}_{I}$, where the interaction term ${\hat{H}}_{I}$ is switched on for a finite time interval.
- In a *mediated interaction* channel, there exists a mediating system described by the Hilbert space $H_{M}$. Both emitter and receiver interact through the mediator, but not directly with each other. Then, the Hamiltonian is $\hat{H}={\hat{H}}_{A}+{\hat{H}}_{B}+{\hat{H}}_{M}+{\hat{H}}_{AM}+{\hat{H}}_{BM}$, where ${\hat{H}}_{AM}$ describes the interaction of the emitter with the mediator and ${\hat{H}}_{BM}$ describes the interaction of the receiver with the mediator. These interactions are usually viewed as permanent, i.e., that they cannot be switched on or off.

If $\hat{U}$ is the unitary evolution generated by this Hamiltonian, then

(2) $$\begin{array}{c} C\left[{\hat{\rho }}_{e}\right]=Tr_{H_{A}⊗H_{M}}\left(\hat{U}\left({\hat{\rho }}_{e}⊗{\hat{\rho }}_{0}⊗{\hat{\rho }}_{M}\right){\hat{U}}^{†}\right), \end{array}$$

where now ${\hat{\rho }}_{M}$ is the initial state of the mediator, typically taken as the ground state of the mediator’s self-Hamiltonian ${\hat{H}}_{M}$.

The emitter and receiver are often spatially localized and separated by a finite distance *L*, in which case the issue of signal propagation arises. Direct interactions lead to faster than light-signal, while it is expected that mediated transmission through local interactions respects causality.

### 2.2. Problems with Treating QFT as Quantum Channel

Quantum communication protocols with photons often employ the electromagnetic (EM) field as a transmission channel. For example, telecommunications from radios to mobile phones treat the classical EM field as a channel for the transmission of information. In these cases the classical EM field configurations are identified with specific states of the quantum EM field. Therefore it is tempting to view quantum fields that mediate an interaction as defining informational channels. However, the language of channels does not capture the full physics of interacting quantum field theories (QFT). The reasons are the following.

- A consistent theory of interactions exists only if receiver and emitter are also treated by QFT. However, then, the Hamiltonian of the system cannot be defined on a tensor product Hilbert space $H_{A}⊗H_{B}⊗H_{med}$, as in a mediated interaction channel. This statement follows from classic theorems by Haag [1] and Hall and Wightman [2]. This means that *the degrees of freedom of the field are intertwined with those of the emitter and the receiver,* in a way that it is impossible to disentangle. The root of this problem is phenomena such as vacuum polarization—it is impossible to describe the field vacuum as a factorized state or even an entangled state in a factorized single state.

This implies that we must use dressed states for the emitter and the receiver, i.e., the mediator degrees of freedom affect the available states of receiver and emitter. The sharp separation is possible only if we work to lowest order in perturbation theory, thus avoiding all Feynman diagrams with loops that mix the degrees of freedom of the emitter/receiver with those of the mediating field. The treatment of the field as an information channel does not make sense beyond this regime.

- The notion of a quantum channel originates from Quantum Information Theory (QIT), which has mainly been developed in the context of non-relativistic quantum mechanics, a small corner of full QFT. *Current QIT is problematic when basic relativistic principles—both special and general—such as causality and covariance, need be accounted for*. A relativistic QIT that expresses all informational notions in terms of quantum fields is currently missing, largely because of difficulties in formulating a comprehensive QFT theory of measurement [3].

Here, we point out one particular problem, namely, the fact that the notion of a localized quantum system in QFT leads to conflicts with causality. This is important for the consistent definition of localized receivers and emitters in QFT. This result is evidenced by a number of theorems, for example, by Malament [4], Schlieder [5] and Hegerfeldt [6]. These works show that the natural definitions of localized observables together with Poincaré covariance and energy positivity conflict the requirement of relativistic causality.

The most well known set-up where localization appears to contradict causality is *Fermi’s two-atom problem*. In the first analysis of a quantum field as a mediated interaction channel, Fermi analyzed the interaction of two remote atoms through the quantum EM field [7]. He assumed that at time t=0, atom A (emitter) is in an excited state and atom B (receiver) in the ground state. He asked when B will notice a signal from A and leave the ground state. In accordance with Einstein locality, he found that this happens only at time greater than *r*. It took about thirty years for Shirokov to point out that Fermi’s result is an artifact of an approximation [8]. Several studies followed with conclusions depending on the approximations used. Eventually, Hegerfeldt showed that non-causality is generic [9,10], as it depends solely on energy positivity and on the treatment of atoms as localized in disjoint spatial regions.

- *The naive idea of a field as an object that mediates interaction is insufficient* to describe the actual theories of mediating interactions, namely, gauge field theories. The reason is that it does not take into account the presence of constraints, which are a consequence of the gauge symmetry. The same issue appears in the treatment of gravity.

In classical field theory, a constraint means that some degrees of freedom that appear in the Lagrangian formulation are either slaved to other degrees of freedom, or pure gauge in the sense that they do not affect the properties of the system. In other words, they are not true degrees of freedom that propagate through the equations of motion, and they do not represent physical observables.

When quantizing a constrained field theory, probabilities must be expressed solely in terms of true degrees of freedom. All major approaches to quantization end up with a Hilbert space $H_{phys}$, where true degrees of freedom live. The state of the field is given by vectors / density matrices on $H_{phys}$.

For example, in the Hamiltonian treatment of the electromagnetic field, the EM potential can be split in four components: the scalar part $A_{0}$, the longitudinal component $A_{i}^{L}$ and the transverse component $A_{i}^{T}$. The conjugate of $A_{0}$ vanishes, while the conjugate $E_{i}^{L}$ of $A_{i}^{L}$ is slaved to the matter charge density ρ through the Gauss constraint ∇·E=ρ. Then, $A_{0}$ and $A_{i}^{L}$ are pure gauge variables, and the true degrees of freedom consist of $A_{i}^{T}$ and their conjugate momenta $E_{i}^{T}$. The Hamiltonian for charged matter interacting with the EM field is

(3) $$\begin{array}{c} H=H_{mat}+H_{EM}+\frac{1}{2}\int d^{3}xd^{3}x^{\prime }\frac{\rho \left(x\right)\rho \left(x^{\prime }\right)}{|x-x^{\prime }|}+\int d^{3}xj\cdot A^{T}, \end{array}$$

where $H_{mat}$ is the self-Hamiltonian for matter, $H_{EM}$ the self-Hamiltonian of the EM field, and j is the current density. The last term, the Coulomb interaction, follows from the Gauss law constraint. It is non-local, and it depends solely on the matter degrees of freedom through the charge density ρ. Note that the transverse component $A^{T}$ of the EM potential is a non-local functional of the full potential, so the corresponding term in the Hamiltonian is also non-local.

When quantizing all variables in Equation (3) are promoted to operators. In a perturbative treatment of the EM interaction, we employ the Hilbert space $H_{mat}⊗H_{EM}$, where $H_{mat}$ describes the matter degrees of freedom and $H_{EM}$ describes the field degrees of freedom. On this Hilbert space a Hamiltonian that includes all terms in Equation (3) but the last is in principle well defined. The last term describes the interaction of photons with matter, and it can be implemented perturbatively. It is important to emphasize that the Coulomb term is defined as an operator of $H_{mat}$, since it depends only on the charge density operator $\hat{\rho }\left(x\right)$. The latter is well defined modulo proper regularization.

Hence, when splitting the matter Hilbert space $H_{mat}$ as $H_{A}⊗H_{B}$ in terms of the emitter *A* and the receiver *B*, we obtain

(4) $$\begin{array}{c} \hat{H}={\hat{H}}_{A}+{\hat{H}}_{B}+{\hat{H}}_{EM}+\int d^{3}x{\hat{j}}_{A}\cdot {\hat{A}}^{T}+\int d^{3}x{\hat{j}}_{B}\cdot {\hat{A}}^{T}+\int d^{3}xd^{3}x^{\prime }\frac{{\hat{\rho }}_{B}\left(x\right){\hat{\rho }}_{A}\left(x^{\prime }\right)}{|x-x^{\prime }|}. \end{array}$$

This means that the Hamiltonian contains an interaction of the two subsystems by the two terms: the Coulomb term that causes direct non-local interaction between the two subsystems, and the interaction that is mediated by photons. It may be tempting to identify the former with a *direct channel* and the latter with a mediated channel, except for the fact that the former term cannot be switched off. The Coulomb interaction between the subsystems is always present; its expectation is non-zero even in the absence of photons. The Coulomb term affects the preparation of the system, and it may not allow for the preparation of an initial state that is uncorrelated. The presence of the Coulomb term is a consequence of the gauge symmetry of EM: it leads to constraints that define *instantaneous* laws in contrast to the causal dynamical laws of time evolution of the true degrees of freedom of the system.

For the reasons above, we believe that the treatment of relativistic interactions in the language of information channels is justified only as an approximation for specific tasks. Otherwise, it should be taken as a simile with a restricted domain of validity, and certainly not as an expression of fundamental physics.

### 2.3. Implications for Gravity

The analysis for the EM field given previously can also be carried out for gravity in the weak coupling limit, where we treat the gravitational field as small perturbations around some background geometry, the simplest being the Minkowski spacetime. The Hamiltonian in the Arnowitt–Deser–Misner gauge is a sum of three terms [11,12]

(5) $$\begin{array}{c} H_{ADM}=H_{matt}+H_{gsi}+H_{GW}, \end{array}$$

where $H_{GW}$ is the Hamiltonian for gravitational waves, including a term for the interaction of gravitational waves with matter, and $H_{gsi}$ is a non-local interaction term (“*gsi*” stands for gravitational self-interaction). In the non-relativistic regime

(6) $$\begin{array}{c} H_{gsi}=-\frac{1}{2}\int d^{3}xd^{3}x^{\prime }\frac{\mu \left(x\right)\mu \left(x^{\prime }\right)}{|x-x^{\prime }|} \end{array}$$

where μ(x) is the mass density. The self-interaction term $H_{gsi}$ originates from the Poisson equation for the gravitational potential

(7) $$\begin{array}{c} \nabla^{2}\phi =-4\pi \mu , \end{array}$$

which is obtained as a *constraint* in the Hamiltonian analysis of the weak-field, non-relativistic limit of GR. The gravitational potential ϕ is a specific component of the three-metric tensor. Equation (8) implies that ϕ is not an independent variable in the Hamiltonian theory, rather it is slaved to the mass distribution.

The usual quantization procedure for weak gravity is to quantize the mass degrees of freedom separately from the gravitational degrees of freedom (gravitons), and to introduce the coupling through perturbation theory. The resulting theory should be finite at tree level, and work as an effective QFT for graviton physics. This procedure presupposes that quantization commutes with the weak-gravity limit. This is a plausible but highly-non trivial assumption of perturbative quantum gravity. If the two operations do not commute, the gravitational part of (5) should not be treated as an effective QFT with a well defined graviton vacuum.

The key point here is that the nature of the term $H_{gsi}$ is not affected by quantization hypotheses about the gravitational field, because it is a functional solely of the matter degrees of freedom. If matter is quantized, then the matter Hamiltonian must include ${\hat{H}}_{gsi}$, because the latter is expressed in terms the mass density for matter. Then, Equation (8) holds at the level of operators,

(8) $$\begin{array}{c} \nabla^{2}\hat{\phi }=-4\pi \hat{\mu }, \end{array}$$

where $\hat{\phi }\left(x\right)$ is an auxiliary operator constructed from the quantum mass distribution $\hat{\mu }\left(x\right)$. Obviously, if we purport to view the Newtonian interaction between two remote masses given by $H_{gsi}$ as a channel, this channel will be direct.

Many authors have tried to view Newtonian gravity as a fundamentally classical channel. This is only possible if Equation (8) fails to hold for a quantum mass distribution $\hat{\mu }\left(x\right)$. Then, ϕ is a classical variable that is only partially correlated with the mass density. For example, in the alternative quantum theories based on the Newton–Schrödinger equation, the Poisson equation takes the form $\nabla^{2}\phi =-4\pi \langle \hat{\mu }\rangle$, where the expectation value is taken with respect to the quantum state. Then, the interaction Hamiltonian of the system is

(9) $$\begin{array}{c} H_{gsi}=-\int d^{3}xd^{3}x^{\prime }\frac{\mu \left(x\right)\langle \hat{\mu }\left(x^{\prime }\right)\rangle }{|x-x^{\prime }|}, \end{array}$$

i.e., it depends on the quantum state. This leads to a non-linear equation for a wave function, similar to Hartree’s equation governing a large number of particles, which is often invoked in atomic and nuclear physics. (Strictly speaking, the Hartree wavefunction is not a quantum state of the *N*-particle system, but a variational parameter in the mean-field approximation—see, for example, Ref. [13]). The pitfall is to assert that this evolution equation holds even for single or a few individual particles, as many proponents of the Newton–Schrödinger equation do [14,15].

In this approach, Newtonian gravity is not a direct interaction channel. The potential is essentially a stochastic variable correlated with the mass density $\mu_{1}$ of one subsystem; this potential generates a classical stochastic force for the other subsystem. This idea is made explicit by the Kafri–Taylor–Milburn (KTM) model [16] and its significant upgrade by Diosi and Tilloy (DT) [17], where gravity is modeled by a classical noisy channel. Effectively, this model amounts to the continuous measurement of the mass density $\mu_{1}$ of one subsystem [18], and a classical stochastic measurement record $J_{1}$ that acts as a classical control force on the other mass. Again, ϕ in Equation (8) is treated as a stochastic classical variable that is correlated with (but not slaved to) the mass density (which is fully quantum). Note that the original KTM model involves a continuous measurement of particle positions, and mainly focuses on deviations from an equilibrium configuration. The TD model considers the measurement of the mass density, and as such it provides a broader generalization of KTM’s main concept. However, they are distinct models, as the restriction of the TD model to the regime where the KTM model applies gives different results.

It is important to emphasize that there is no fundamental justification for the treatment of the gravitational potential ϕ as a stochastic variable, except for the plain desire to define a classical channel for the gravitational interaction. The idea that Newtonian gravity can be described by a classical/stochastic interaction channel contradicts important *facts* about the gravitational field. In particular, it contradicts our understanding that in weak gravity, save for gravitational waves which are the true dynamical degrees of freedom, gravity is completely slaved to the distribution of matter. Any theory that violates this property must explain the physical origin of this violation. After all, Newtonian gravity is embedded in General Relativity, so any modification of the former implies invariably a modification in the later. In particular, the slaving of the potential to matter via Poisson equation is a constraint of General Relativity, which expresses the invariance of the theory under time reparameterizations, a fundamental symmetry of GR.

Note that the KTM model and some of its variations are incompatible with atomic fountain data [19]. However, our critique about incompatibility with GR applies to all possible implementations of classical channels for Newtonian gravity.

The motivation for proposals by KTM and TD is the idea that gravity, i.e., spacetime geometry, is fundamentally classical, and that it should not be quantized. Along this way of thinking, quantum matter coupled to classical gravity invariably involve stochastic behavior for the gravitational dynamical variables. However, if one agrees that classical gravity is governed by GR, then at the weak gravity level, the only dynamical variables are gravitational waves; the Newtonian force is certainly not. The assumption of a quantum- classical coupling of matter with Newtonian gravity, which involves no true degrees of freedom for the gravitational field, is inexplicable from the perspective of GR.

There is also the possibility that a fundamental quantum to classical-stochastic coupling of matter to geometry could lead to a different identification of degrees of freedom at the weak coupling limit. One example is the post-quantum theory of gravity of Refs. [20,21], in which classical gravity is coupled to quantum matter. This theory is characterized by a symmetry of spatial diffeomorphisms (rather than spacetime diffeomorphisms, as required by General Covariance). However, the structure of constraints in this theory, their relation to symmetries and their physical interpretation need to be resolved clearly, as they pertain to the identification of the true degrees of freedom at the relevant regime of weak gravity.

In the language of information channels, GR predicts unambiguously a direct interaction channel between two separated quantum mass distributions, obtained by adding the quantum version of the operator $H_{gsi}$ in the Hamiltonian for quantum matter. This channel will lead to phenomena such as entanglement generation [22,23] and Rabi-type oscillations [24]. However, since this channel is direct, the observation of such phenomena says nothing about the nature of the true degrees of freedom of the gravitational field. As the latter effectively decouple from the Newtonian interaction at the level of weak gravity, one cannot make any statement about their nature, classical or quantum, from the observation of gravity-induced entanglement generation.

The observation of gravity-induced entanglement certainly rules out models in which Newtonian gravity corresponds to a classical/stochastic channel, or models with a classical mediator for Newtonian gravity. However, as explained here, these models are rather implausible from the perspective of gravity theory. A more plausible class of models involves a classical/stochastic interaction mediated by the true degrees of freedom of gravity (gravitational waves), together with the direct-channel interaction of Newtonian gravity, in the vein of the Anastopoulos–Blencowe–Hu model of gravitational decoherence [25,26]. These models are fully compatible with gravity-induced entanglement in the Newtonian regime, and they provide an explicit counter-example to claims of proving the quantumness of gravity from experiments with Newtonian gravity.

Ref. [27] presents an argument why a mass in a spatial superposition that interacts gravitationally with a test mass leads either to faster-than-light signalling or violation of quantum complementarity, unless one takes into account vacuum fluctuations of the gravitational field and the emission of gravitational radiation. However, a close reading of the argument shows that vacuum fluctuations need not be quantum, and that the restoration of quantum complementarity only requires a decoherence mechanism—spontaneous emission of discrete quanta being only one of many possible scenarios. Hence, the arguments of Ref. [27] are not stringent enough to rule out theories in which the true degrees of freedom of gravity are treated as a classical stochastic field that causes decoherence to quantum matter. However, these are properties that any mathematically consistent quantum-to-classical coupling must have anyway: mathematically consistent couplings of quantum to classical variables typically induce non-unitary evolution and decoherence to the quantum system and noise to the classical system [28,29,30,31]. Models for quantum-to-classical coupling without decoherence and noise typically lead to the violation of positivity, i.e., negative probabilities.

The same point applies to the extended analysis presented in Ref. [32]. There, it is also argued that in the thought-experiment of Ref. [27], there is no clear distinction between entanglement mediated by the Newtonian gravitational field of a body and entanglement mediated by on-shell gravitons emitted by the body. This may be the case for the logical coherence of their analysis, but there is a fundamental difference between Newtonian force and (on-shell, not virtual) graviton interactions, the former slaved to matter by constraints, the latter carries the true dynamical degree of freedom. Thus, only in the detection of gravitons can one ascertain the quantum nature of (perturbative) gravity. In particular, in the proposed experiments [22,23] entanglement is caused specifically by the Newtonian force, and not by on-shell gravitons.

Cosmology is an interesting arena to check out different ideas about the quantum nature of gravity. Primordial density contrasts are affected by gravitational perturbations in both the scalar and the tensor sectors. However, gravitational waves in the transverse-traceless gauge belong to the purely tensor sector which is decoupled from these other sectors tied to the matter [33]. The decoupled tensor sector (the ‘B modes’), if detected, could provide evidence that perturbative gravity is quantum. This is consistent with our assertion, namely, only the transverse-traceless modes carry the dynamical degrees of freedom of gravity, and only through them would the quantum nature of gravity show, not from the pure gauge tied to matter. However, notice this is only *perturbative* quantum gravity centering on the existence of gravitons. Gravitons could be viewed as the quantized collective modes of excitation (such as phonons) of spacetime if spacetime is considered as the low energy effective theory (long wavelength hydrodynamical limit) of quantum gravity proper. These theories, e.g., fundamental string or quantum loop theory, operative at the Planck scale, which attempt to decipher the basic constituents (the atoms) of spacetime are fundamentally different from perturbative gravity which can exist at today’s low energy.

### 2.4. Event Formalism and Closed Timelike Curves

The event formalism [34,35] is an alternative quantum theory that predicts photon disentanglement in the presence of gravity. It is built under the assumption that the operators representing observables at different points along a particle’s geodesic are required to commute with one another. This requirement implies a strong and rather ad hoc modification to the properties of quantum fields. This formalism stems from the analysis of [36] in finding possible unitary solutions to a quantum gravity information paradox. It is based on the treatment in [37] of closed timelike curves in the context of quantum computation.

We want to make two points in relation to these models. First, the modification stems from arguments that are based on quantum computation in terms of quantum circuits, not on a QFT analysis. As [37] asserts, such computational circuits are universal in the sense that they can simulate the behavior of finite quantum systems. This means that they can simulate the behavior of a *finite* number of modes of the quantum field. However, the simulation of a QFT can be highly non-local, as, for example, when simulating fermion fields with qubits [38]. There is no guarantee that the computational degrees of freedom are localized on spacetime, and that they are subject to the usual analysis of locality and causality.

More importantly, the idea that closed timelike curves exist without accompanying quantum gravity effects is highly implausible from the perspective of gravity theory. This strongly contradicts the well-motivated *chronology protection conjecture* [39] which asserts that the laws of physics, including quantum phenomena, do not allow for the appearance of closed time-like curves. Chronology protection is valid also for semi-classical gravity [40]. All this strongly suggests that closed timelike curves can emerge only as Planck scale quantum gravity effects (like Wheeler’s spacetime foam [41]), if at all. Hence, any phenomena stemming from closed timelike curves stretch the capabilities of current experiments more than 20 orders of magnitude, so there is no surprise of null findings [42].

## 3. Problem 2: Quantum Processes/Fluctuations Cannot Be Replaced by Classical Stochastic Processes/Noises

In this and in the next section we turn our attention to the relation of quantum fluctuations and classical stochastic processes. Noise is often introduced and stochastic equations invoked in many alternative quantum theories for some specific purposes, such as in explaining why we do not see quantum effects in the macroscopic world (e.g., the stochastic Schrödinger equation in [43,44]). We begin by showing how fluctuations, often at the Planck scale, have been proposed as the source of gravitational decoherence, and what goes wrong in doing so. Gravitational decoherence is a good case study as it involves all three aspects: gravity, quantum field and quantum information.

### 3.1. Fluctuations as Sources of Gravitational Decoherence—What Is Missing or Misleading

In many proposed models, gravitational decoherence is a consequence of fluctuations that are assumed to originate from Planck-scale physics. The specific mechanism varies. Fluctuations may be induced by some fundamental imprecision in the measuring devices (starting with clocks and rulers) [45,46], or by uncertainties in the dynamics [47,48], or by treating time as a statistical variable [49], to give some examples.

Obviously, any model that involves Planck scale physics must make very strong assumptions. However, we find assumptions that Planck scale uncertainties can be modeled by classical stochastic processes rather implausible. The modeling of uncertainties by classical noise may work for randomness at the macroscopic scale where quantum properties do not play a role. In contrast, quantum uncertainties are different in nature as they involve non-localities and correlations with no analogues in the classical theory of stochastic processes.

To explain this point, we note that the limitations posed by the Planck length are not a priori different from those placed by the scale $\sqrt{\hbar /c^{3}}e/m_{e}$ in quantum electrodynamics, where *e* is the electron charge and $m_{e}$ is the electron mass. At this scale quantum field effects are strong, and the fluctuations from these effects are fully quantum. Any effect they cause at low energy is also inherently quantum. One needs to specify the conditions (e.g., Gaussian systems) or the regime for the quantum field, and justify the means by which they could be treated such as classical fluctuations described by a stochastic process. In particular, the effects of the fluctuations of the electromagnetic field at low energies ($E<<mc^{2}$) have been well studied. It has been shown that the ‘noise’ induced by these fluctuations is non-Markovian and does not cause significant decoherence effects in the microscopic regime [50,51,52]. In other words, the coherence of the electromagnetic field vacuum does not allow for the a priori generation of classical (i.e., decohering) fluctuations in the quantum motion of the particle. The assumption that the gravitational field exhibits a different behavior is completely ad hoc, with no justification unless one postulates that *gravity is fundamentally classical*.

### 3.2. Classical Stochastic Processes or Noises Miss Out Important Information in Quantum Theories

When encountering the effects of quantum fluctuations, a common practice of authors who know enough about classical stochastic processes is to assert or assume that they can be replaced by classical stochastic processes. Without a rigorous proof, this substitution is unwarranted. Making this jump could also be dangerous, if one does not even know what has been left out or what can be messed up in this substitution. Important quantum features are ignored which bear on basic quantum information issues such as entanglement and coherence.

In some cases, quantum fluctuations of an observable may be replaced by a classical stochastic process *after coarse-graining*. This is possible if the coarse-grained observable satisfies specific consistency conditions [53,54]. Then, it can be rigorously shown that the measurement outcomes for this observable can be modeled by a stochastic process [55]. To this end, it is necessary that all specific quantum properties of the fluctuations are suppressed by coarse-graining.

Hence, except for specific limiting regimes, quantum processes cannot be replaced by classical stochastic processes. We shall show that even in cases of the closest proximity, namely, for Gaussian systems, where the Wigner function remains positive definite and has the same form as a classical distribution function, there are subtle and important differences: a quantum theory contains more information than the corresponding classical stochastic ones.

The statistical properties of a classical stochastic field can be described by a classical distribution functional of the field variable and its conjugate momentum over an infinite-dimensional phase space spanned by the canonical pair. The distribution function of the classical stochastic field is often misconstrued as the equivalent of the quantum Wigner function. Even for Gaussian systems it is not completely true that its quantum behavior is identical to the classical: Wigner functions carry more information than what is in the corresponding classical probability distributions. This is no surprise because Wigner function carries the full equivalent information contained in the density matrix.

Can a classical stochastic field act as a functional surrogate of a quantum field? This old issue is revisited in a recent paper by Hsiang and Hu (HH) [56], where a conduit for Gaussian systems is built connecting a full quantum field-theoretic treatment and those using the probability distribution of classical stochastic fields. Since the bridging protocol is the source of a great deal of confusion, HH examine the conditions for the two theories to be connected, while paying special attention to the ability of the latter to preserve essential quantum properties. They conclude that the information contained in *classical stochastic field theory is far from complete*, e.g., it still needs inputs from the two-point functions of the quantum field to yield the stochastic counterparts. Even though from certain angles both look formally identical, the theoretical frameworks they are based on are fundamentally different.

We list the key points from their findings, beginning with the obvious question: (A) How is quantum non-commutativity represented? In quantum theory, the Wigner function corresponds to a *fully symmetrized ordering*. HH demonstrate that for those physical quantities of interest which can be expressed in terms of the covariance matrix elements, which are the expectation values of the canonical operators in *symmetrized ordering*, the stochastic field approach seems to work. However, this is unlikely for other ordering choices. In the classical stochastic field approach, different distribution functions may be needed to account for different operator orderings in quantum theory. (B) The Wigner functions for the more general *non-Gaussian configurations* are not positive-definite and the correspondence with classical distribution functions breaks down. Crucial for *quantum information issues*, (C) classical field theory and its stochastic renditions cannot account for quantum entanglement, in which the nature of quantum state plays a pivotal role.

We can see a glimpse of this from (D) the differences between the classical Shannon entropy and the quantum von Neumann entropy. The Shannon entropy associated with classical distributions has the property of monotonicity. That is, given the combined systems *A* and *B*, we have $S_{s}\left[A+B\right]\geq \max\left\{S_{s}\left[A\right],S_{s}\left[B\right]\right\}$ for the Shannon entropy, where $S_{s}\left[A\right]$ denotes the Shannon entropy of the subsystem *A*. On the other hand, for the von Neumann entropy S, the closest property to the classical monotonicity is the theorem of Araki–Lieb triangle inequality:

(10) |S[A]−S[B]|≤S[A+B]≤S[A]+S[B].

The second group of inequalities is known as the subadditivity inequality for von Neumann entropy, and holds with equality if and only if systems *A* and *B* are uncorrelated, that is, $\varrho^{AB}=\varrho^{A}⊗\varrho^{B}$. HH has shown (in the case of particle creation, where subsystems A,B represents the ±k modes of the pair) that S[A,B]=0 but S[A]=S[B]>0. The entropy of a subsystem is larger than the entropy of the combined system. These properties are broadly known, often invoked in the discussions of entanglement entropy.

The message we wish to convey is, as a matter of principle, one should seek a quantum field description where the effects of quantum fluctuations are fully incorporated. Furthermore, if or when a classical stochastic process may provide an adequate description of the quantum field, specify the conditions and spell out the limitations.

## 4. Problem 3: How Are Semiclassical and Stochastic Theories Related to Their Quantum Origins? How Does Noise Enter?

The second question raised here is easier to answer. If one permits noise to be added by hand, there is always a stochastic counterpart to any theory. This practice has turned into an unquestioned habit in many situations, likely attributable to the way how we first encounter a stochastic equation, such as the Langevin equation: “just add a noise term as source driving the Newton equation”. Voila! Only later did we learn that in doing so a closed system where the dynamics is unitary is changed to an open system following nonunitary dynamics. We need to identify an environment which our system interacts with and find out the coarse-graining measures which produce the noise chosen, which drives the system. Furthermore, we need to make sure the system’s dissipative dynamics and the environmental noise observe a self-consistency requirement, such as governed by a fluctuation-dissipation relation. Many proposers of alternative quantum theories like to introduce a noise source, turning, say, a Schrödinger equation into a stochastic Schrödinger equation, which serves their specific purposes. Regrettably, rarely do these authors explain where their noises come from, their physical meanings, and whether turning a unitary equation into a stochastic equation generates any mathematical conflicts with, or compromises the theoretical foundation of, the established theories.

There is one situation we know of and have worked on, where a classical stochastic source (or noise) can be derived as a full representation of quantum field fluctuations, for systems and environments being all Gaussian. We shall explain this in the next subsection.

Let us now turn to the first question: How are semiclassical and stochastic theories related to their progenitor quantum theories? For this discussion, it is useful to keep in mind the four distinct levels of theoretical structures: quantum, stochastic, semiclassical and classical, in the order of decreasing information contents. Every lower level theory is a limiting case of the higher level theories, with the quantum theory as their source. Note in particular the stochastic level here is different from theories obtained by putting a noise in by hand. Instead, stochastic follows quantum only because it is a genuine derivative of the quantum theory under specific conditions. It is useful to review this four-level structure so that as one sees some stochastic source entering one can identify it in the right place and decide whether it can be derived or just introduced in an ad hoc manner, a big difference.

### 4.1. Semiclassical Theory as Large *N* Limit of Quantum Theory

The relation between semiclassical and quantum theory is a well-studied subject ever since quantum theory was established, and there is almost a semiclassical theory for every quantum theory which came into being, constructed from the corresponding longer existing and more familiar classical theory: quantum theory of radiation, quantum chaos, etc. There are also cases where the quantum theory is well established but a lesser theory is introduced with the help of experimental input in order to bypass the difficult calculations, Lifshitz’s stochastic electromagnetic theory is a good example, with monographs devoted to it [57,58]. Semiclassical theory plays a special role for gravity because of the lack of a *bona fide* quantum theory of (nonperturbative) gravity. There, gravity is kept classical, described by Einstein’s theory of general relativity (GR), while matter is described by quantum field theory (QFT), both theories have been tested to the utmost degree, the best we have for the description of spacetime and matter.

Currently we see some increased interest in semiclassical gravity theory among researchers of gravity, quantum fields and quantum information, and with it, also misconceiving or misconstruing what a correct SCG theory should be. Let us start with the popular Newton–Schrodinger Equation (NSE). On surface it looks like the weak field limit of general relativity and the nonrelativistic limit of the Klein–Gordon or Dirac equation. However, it is not. Single or few particle NSE cannot be derived from GR+ QFT—see [14] for details or [59] for a summary.

Semiclassical theory makes sense as the large *N* limit of the progenitor quantum theory. The semiclassical Einstein equations, the Einstein tensor $G_{\mu \nu }=8\pi G\langle {\hat{T}}_{\mu \nu }\rangle ,$ where ${\hat{T}}_{\mu \nu }$ is the stress energy tensor of quantum matter field, are only meaningful for *N*-particle quantum states with N>>1, where the relative strength of fluctuations is suppressed by a factor of $N^{-1/2}$. Theories using the Newton–Schrödinger equation and theories that treat the Einstein–Klein–Gordon, Einstein–Dirac equations or the Moller–Rosenfeld Equation [60,61] in a prima face, abridged or altered way, are not compatible with GR+QFT. The crucial point is in the coupling between classical gravity and quantum fields, such as stated in the Wald axioms [62,63], and the requirement of self-consistency. The large *N* limit of (perturbative) quantum gravity has been shown [64] to be a pathology-free and self-consistent semiclassical gravity theory based on the relativistic semiclassical Einstein equations. (Please refer to the four levels, from 0 to 3, of “semiclassical gravity” theories in [59]. The proper ones to use are Levels 2 and 3 (p. 7), what are referred to as ‘relativistic semiclassical gravity’ and ‘stochastic semiclassical gravity’).

In the next subsection, after we have introduced the stochastic semiclassical gravity theory [65,66] based on the Einstein–Langevin Equation [67], we shall see that the relativistic semiclassical gravity theory is obtained after performing a stochastic average over the noise which describes the quantum fluctuations of matter field. In this sense relativistic semiclassical gravity is a relativistic “mean field” theory.

### 4.2. Stochastic Semiclassical Theory: Noise Can Be Defined for Quantum Fluctuations in Gaussian Systems

In the beginning of this section we mentioned that noise in a stochastic equation is often put in by hand. However, one can define noise of a Gaussian quantum environment in a mathematically rigorous manner, namely, by way of the Feynman-Vernon [68] Gaussian functional identity. Because of the way how this noise is derived, it is best to keep in mind its quantum origin, namely, that it is only a stochastic classical source *representation* of quantum fluctuations, its true nature being fully quantum mechanical. In this way one can distinguish from ad hoc noises attached to a quantum equation such as in the stochastic Schrödinger equation, or put in by hand to some lower (classical or semiclassical) level equations.

Let us examine stochastic semiclassical gravity in the large *N* vein. When the quantum gravity sector is coupled to a large number *N* of matter (free) fields, the lowest-order contribution in a 1/N expansion produces the semiclassical Einstein equations. When using large *N* the cut-off scale is shifted to the rescaled length. At the lowest order the fluctuations of the metric are suppressed but all matter loops are included. In the same vein, stochastic gravity results from taking the next-to-leading order in the 1/N expansion of quantum gravity coupled to *N* matter fields. Whereas in semiclassical gravity the fluctuations of the metric are suppressed, here in stochastic gravity, metric fluctuations are of linear order, while graviton loops or internal graviton lines are sub-leading in comparison to matter loops. Semiclassical and stochastic gravity as effective theories in the large *N* context are discussed in Chapters 9 and 10 of [66].

Harking back to the issue of gravitational decoherence by stochastic processes at the Planck scale, we remarked that models which introduce noise or invoke fluctuations which are intrinsically classical have pathologies. The proper way to introduce stochasticity in spacetime which respects both general relativity and quantum field theory is through the metric fluctuations induced by the fluctuations of quantum matter fields (via the noise kernel which is the correlator of the stress energy tensor of quantum matter field, see, e.g., [69,70]) by seeking solutions to the Einstein–Langevin Equation (ELE) [67,71,72,73,74]. This has been carried out successfully for the Minkowski metric in [75,76], and for inflationary cosmology in [77]. In stochastic semiclassical gravity the noise is fundamentally quantum; the backreaction of a quantum matter field and its fluctuations on the classical geometry and its fluctuations is obtained by solving the ELE which guarantees fully the self-consistency between the quantum matter field and the classical geometry sectors. In fact, the magnitude of metric fluctuations enters as the most stringent criterion of the validity of semiclassical gravity [78,79,80].

Returning now to the low energy limit, where laboratory or space experiments can provide some reality checks, upgrade to a stochastic Newton–Schrödinger equation has been proposed [81,82] with the intention of explaining the origin of noise in some of the alternative quantum theories. Here again, our critiques of the NSE apply. The proper way is to take the weak field nonrelativistic limit of the Einstein–Langevin equation. This limit of stochastic gravity is of special interest as it is the only known consistent theory which can deal with quantum entanglement issues in a gravitational setting, such as in the physics of gravitational cat states [24] (see also Sec. 1.3.4 of [66].)

Above all, in the consideration of gravity and quantum fields, back-reaction self-consistency is the ultimate criterion one needs to respect. The way how these two sectors are coupled, how noise of quantum field is derived, how the quantum expectation values and stochastic averages are taken, how a quantum field and its fluctuations back-react on a classical background spacetime in a self-consistent manner—they are all of crucial importance. Improper treatments or assumptions in the consideration of any of these factors and their related issues can lead to pathologies disallowed in general relativity and quantum field theory.

## 5. Conclusions

Gravity (G) and quantum (Q) are two main old yet still vibrantly blooming branches of physics at the foundations of modern science. In the last two decades the introduction of ideas, practices and ways of thinking in quantum information (I) theory has stimulated new growth in both of these old vines and their crossings, a very welcoming development. At the same time alternative quantum and gravitation theories also begin to appear or resurface in abundance. Because many new experimental proposals for testing these alternative theories or the quantum nature of gravity involve atom or molecules (AM) interacting with light (O), we see an increasing share of the perspectives of the AMO community in discussions of the foundational theoretical issues of these three subjects Q, G, I and their unions. These perspectives include, but are not limited to the following.

(i) Staying in the confines of nonrelativistic quantum mechanics to describe quantum field processes and quantum information;
(ii) Using information channels to describe quantum and gravitational effects in substitution of QFT and GR;
(iii) Introducing classical stochastic sources or processes to mock up quantum field fluctuations or replace quantum field processes, leading to theories with inconsistencies or pathologies.

In this article, we have shown the problems arising from making such assumptions and the pitfalls in such treatments. In relation to point (i), we use the Newton–Schrödinger equation as a case study and recall the problems we pointed out in our earlier papers.

In relation to point (ii), we analyzed the notion of interactions mediated by an information channel and showed that, in general, it is not equivalent to the treatment of interactions by QFT. We gave three reasons. First, QFT is not compatible with the tensor product structure of interaction channels, except as an approximation. Second, the notion of localization and its relation to causality is more nuanced in QFT than in standard QIT. Third, the idea of quantum fields as channels that mediate interaction is insufficient to describe the actual theories of mediating interactions, namely, gauge field theories. Then, we showed that the use of information channels for the description of Newtonian gravity may lead to inconsistencies with general relativity: it ignores the fact that GR predicts that gravity is completely slaved to matter in the Newtonian regime, and has no dynamical content.

In relation to point (iii) we point out that in general one cannot replace a quantum field by a classical stochastic field, or mock up the effects of quantum fluctuations by classical noises, because important quantum features such as coherence and entanglement will be left out, as the nature of quantum states enter into the consideration. The distribution function of a classical stochastic field is often misconstrued as the equivalent of a quantum Wigner function because of their formal resemblance in some cases. However, even for Gaussian systems it is not true that its quantum behavior is completely identical to the classical: Wigner functions, carrying the full information equivalent to that of density matrices, contain more information than in the corresponding classical probability distribution functions. The information contained in a classical stochastic field theory is far from complete, e.g., it still needs inputs from the two-point functions of the quantum field to yield the correct stochastic counterparts, and, how does it address the non-commutativity and the operator-ordering issues? Turning a unitary evolutionary equation into a stochastic equation by adding a noise as source in an ad hoc manner could create problems because often it violates the self-consistency condition such as contained in the fluctuation-dissipation relation.

The Feynman-Vernon functional identity for Gaussian systems provides a consistent way of defining noise for quantum fields. This allows us to link up the semiclassical with the stochastic in the four levels of theoretical structures, from classical to semiclassical to stochastic to quantum. To describe how a theory at a sub-level can be obtained from a higher level, we use *semiclassical and stochastic gravity* theories as illustrations. In a theory of quantum gravity with a large number *N* of matter (free) fields, the lowest-order contribution in a 1/N expansion produces the *semiclassical Einstein equations*. Taking the next-to-leading order in the 1/N expansion of quantum gravity coupled to *N* matter fields yields stochastic gravity. Whereas in semiclassical gravity the fluctuations of the metric are suppressed, in stochastic gravity, metric fluctuations are of linear order with graviton loops or internal graviton lines sub-leading in comparison to matter loops. The proper way to introduce stochasticity in spacetime is through the metric fluctuations induced by the fluctuations of quantum matter fields via the noise kernel by seeking solutions to the *Einstein–Langevin equations*.

## Data Availability

Not applicable.
